# Enhancement of the Processability and Properties of Nylon 6 by Blending with Polyketone

**DOI:** 10.3390/polym13193403

**Published:** 2021-10-03

**Authors:** Tao Zhang, Ho-Jong Kang

**Affiliations:** Department of Polymer Science and Engineering, Dankook University, 152 Jukjeon-ro, Suji-gu, Yongin-si 16889, Gyeonggi-do, Korea; taozhang1214@gmail.com

**Keywords:** nylon 6, polyketone, chain extender, hydrogen bonding, chain branching, chain crosslinking, melt viscosity

## Abstract

Polyketones (PKs) having strong hydrogen bonding properties and a chain extender are used as additives in the melt processing of nylon 6 (PA6). Their effect on the chain structure and properties of PA6 is studied to enhance the processability of PA6 in melt processing. The addition of the chain extender to PA6 increases the melt viscosity by forming branches on the backbone. The addition of PKs results in an additional increase in viscosity through the hydrogen bonding between N–H of PA6 and C=O of PK. The change in the N–H bond FT-IR peak of PA6 and the swelling data of the PA6/PK blend containing a chain extender, styrene maleic anhydride copolymer (ADR), suggest that incorporation of chain extender and PK in the melt processing of PA6 results in physical crosslinks through hydrogen bonding between the branched PA6 formed by the addition of chain extender and PK chains. This change in the chain structure of PA6 not only increases the melt strength of PA6 but also increases randomness resulting in decreased crystallinity.

## 1. Introduction

Polyamide is the most widely used thermoplastic polymer among engineering plastics due to its superior mechanical properties and chemical resistance [1,2,3]. PA6 has generally been used as a textile material substituting natural fibers [4,5], but recently, it is being used widely for automotive parts due to its excellent impact resistance and abrasion resistance [6,7,8]. PA6 automotive parts are generally processed by melt processing such as extrusion or injection molding, and for thermal stability, chain extenders can be used to introduce branches and increase the molecular weight [9,10,11]. Diverse reinforcing materials can also be introduced to prepare nylon composites [12,13]. As the extrusion or injection molding of PA6 is carried out at a relatively high temperatures above 220 °C, chain scission by thermal degradation results in a decrease in the molecular weight [14], and thus, the melt strength and mechanical properties of the product are decreased significantly. The thermal degradation is known to be accelerated by the presence of water in PA6 [15].

To deal with the thermal degradation during melt processing, the following methods have been reported: solid-state polymerization (SSP), where the thermal degradation products can be removed by the introduction of inert gas in a vacuum state to minimize further thermal degradation [16]; and extension of the degraded chain by the introduction of chain extenders containing functional groups, which can react with the diverse end groups produced by the degradation of PA6 [17]. The most widely used chain extenders for PA6 are those that can react with the carboxyl (–COOH) or amino(–NH_2_) groups produced by the thermal degradation of PA6 in the presence of water such as bis caprolactam [18], bislaurolactamdiaryl lactam, or bisoxazoline [19] or those containing anhydride functional groups, which can react with O=C–NH_2_ or –CH=CH_2_ end groups produced by thermal degradation in the absence of water, such as Joncryl ADR [20,21] or epoxy resin [22]. The addition of a chain extender increases the molecular weight of PA6 by branching or partial crosslinking of PA6 through reaction with the end groups produced in the high-temperature melt processing to result in an increase in the melt strength, thus enhancing the processability and overall properties such as mechanical properties.

PA6, poly(ethyl cyanoacrylate)(PECA), PK, etc. with polar groups such as –NH, –OH, and C=O can exhibit dipole–dipole interaction, ion–dipole interaction, and hydrogen bonding in blending. Especially, PK having –C=O groups in the main chain capable of forming strong hydrogen bonds is reported to be very compatible with PA6 in melt processing due to its hydrogen bonding with the –NH bonds of PA6 [23,24,25,26]. Although this hydrogen bonding is expected to affect the chain structure of PA6 and thus the overall properties, there is no report emphasizing this aspect. Direct modification of PK with diamines resulted in a dramatic increase of the tensile strength of PK [27]. It was also found that hydrogen bonding between PK and PECA resulted in interpenetrating networks by which wettability and morphology can be controlled [28].

In this study, enhancement of the melt strength and mechanical properties through the introduction of branches to PA6 is attempted by adding PK, thereby, enhancing the melt viscosity of PA6 in injection molding. The capability of PK to form hydrogen bonds with PA6 in the melt processing of PA6 with chain extender was investigated. The effect on the chain structure of PA6 and the resulting change in the rheological and mechanical properties are studied.

## 2. Experimental

### 2.1. Materials

The PA6 used in this study was Taekwang RV 2.10 (Seoul, Korea), with a melting point of 221 °C, specific gravity of 1.12 g/cm^3^. The PK used as an additive was Hyosung M630A (Seoul, Korea), a terpolymer of carbon monoxide, ethylene, and propylene, with a melting point of 218 °C, MI 6 g/10 min, propylene content of 5.64%, and specific gravity of 1.24 g/cm^3^. The chain extender, multifunctional styrene-acrylic oligomer (Joncryl ADR 4370, Qingdao, China), was purchased from BASF. Antioxidant ZIKA-1010 6683-19-8 was purchased from ZIKO (Anyang-si, Korea) and used to minimize thermal degradation in the melt processing. Tetrahydrofuran (THF, Sigma-Aldrich, Darmstadt, Germany) was used to measure the degree of swelling of PA6 and PA6/PK blend.

### 2.2. Sample Preparation and Reactive Processing

PA6 was vacuum dried at 80 °C for 24 h. to minimize the hydrolytic thermal degradation prior to melt blending. Haake internal mixer (Karlsruhe, Germany) was used to blend dried PA6, ADR, PK, and antioxidant ZIKA-1010 at different ratios. The antioxidant concentration was fixed at 0.2%, and 1, 3, 5 phr ADR and 1, 3, 5, 10 wt.% PK were added, respectively or together, to 40 g PA6 and blended at 220 or 260 °C for 15 min at a stirring speed of 30 rpm. The change in torque with blending time of PA6 with PK and ADR was observed. The blend was compression molded at 220 °C into a 20 mm × 20 mm mold of different thicknesses on a QMESYS QM900A (Uiwang, Korea) and quenched to 4 °C to obtain the samples. Samples (1 mm thick) were prepared for rheological testing, FT-IR and degree of swelling tests, and 0.35-mm-thick samples were prepared for tensile testing.

### 2.3. Characterization

Rotational rheometer AR2000ex (TA Instruments, New Castle, DE, USA) was used to evaluate the rheological properties of the melt. The sample was attached to a 25 mm ETC steel plate, then the complex viscosity and the loss tangent were measured at a rotation speed of 0.1–628 rad/s and 1% deformation at 220 °C.

The crystallinity of the samples (χ) was calculated using the following equation with data obtained on a TA differential scanning calorimeter Q20 (TA Instruments, New Castle, DE, USA) scanning from −50 to 260 °C, at a heating rate of 10 °C/min.

(1) $$\chi =\frac{{\Delta H}_{m}}{{\Delta H^{o}}_{m}}\times 100\%\;$$

where Δ*H_m_*: enthalpy of melting, Δ*H^o^_m_*: enthalpy of melting of pure PA6 (190 J/g) [29].

Infrared spectra of the PA6 and PA6/PK blends were obtained on a Thermo Scientific Nicolet iS10 FT-IR (Thermo Scientific, Waltham, MA, USA) in the ATR mode, in the range 4000–500 cm^−1^ at a resolution of 4 cm^−1^ and scan number of 16.

The degree of swelling in THF was measured to confirm the changes in the chain structure such as partial crosslinking of PA6 on mixing with ADR and PK. A 20 mg (Wd) sample was put in 0.6 mLTHF and sonicated at 50–70 °C for 100 min on a Hwashin Powersonic 410 (Gwangju-si, Korea), then the weight of the sample (Ww) was measured to calculate the degree of swelling (DS%) with the following equation.

(2) $$DS\left(\%\right)=\frac{\mathrm{Ww}-\mathrm{Wd}}{\mathrm{Wd}}\times 100$$

The mechanical properties were evaluated by measuring the tensile strength, Young’s modulus, and elongation at break with 10 mm × 20 mm × 0.35 mm samples on a Lloyd tensile tester LR30K (LLOYD, Cleveland, OH, USA) at a crosshead speed of 10 mm/min.

## 3. Results and Discussion

**Figure 1:**
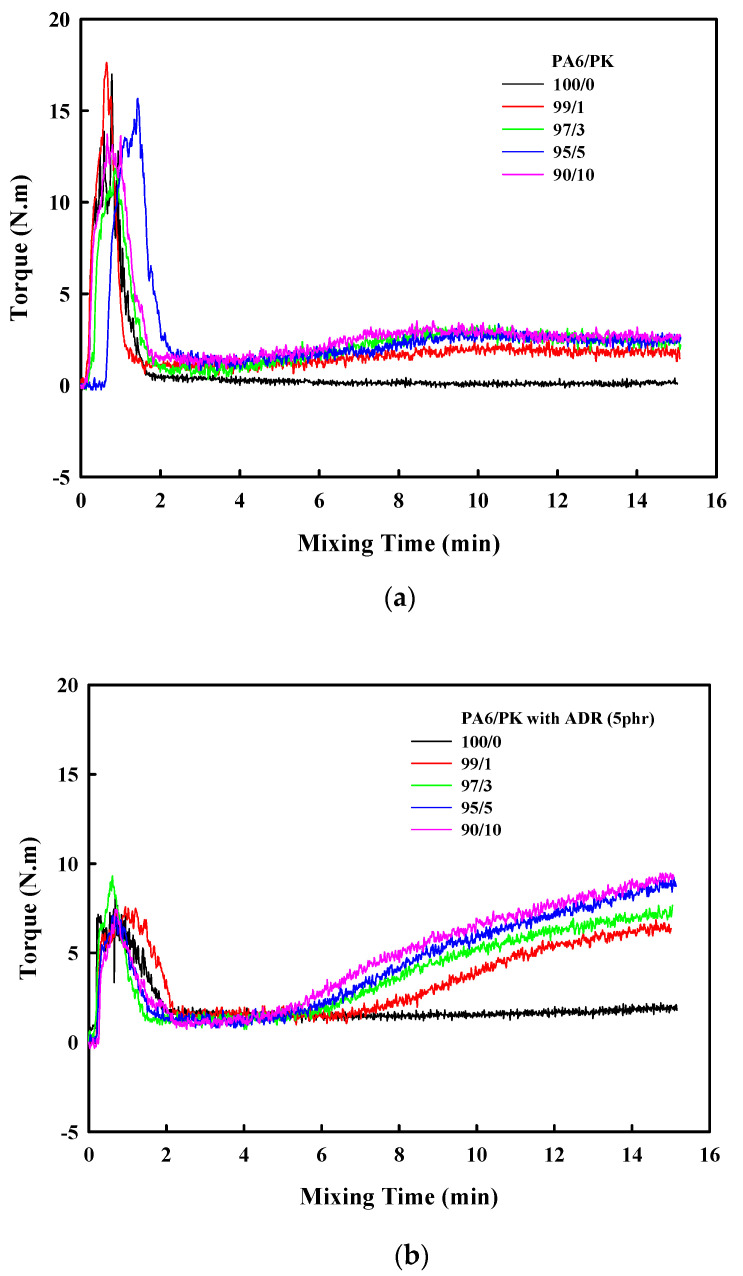
Melt torque as a function of mixing time for PA6/PK blends; (**a**) without chain extender; (**b**) with chain extender(5 phr).

**Figure 2:**
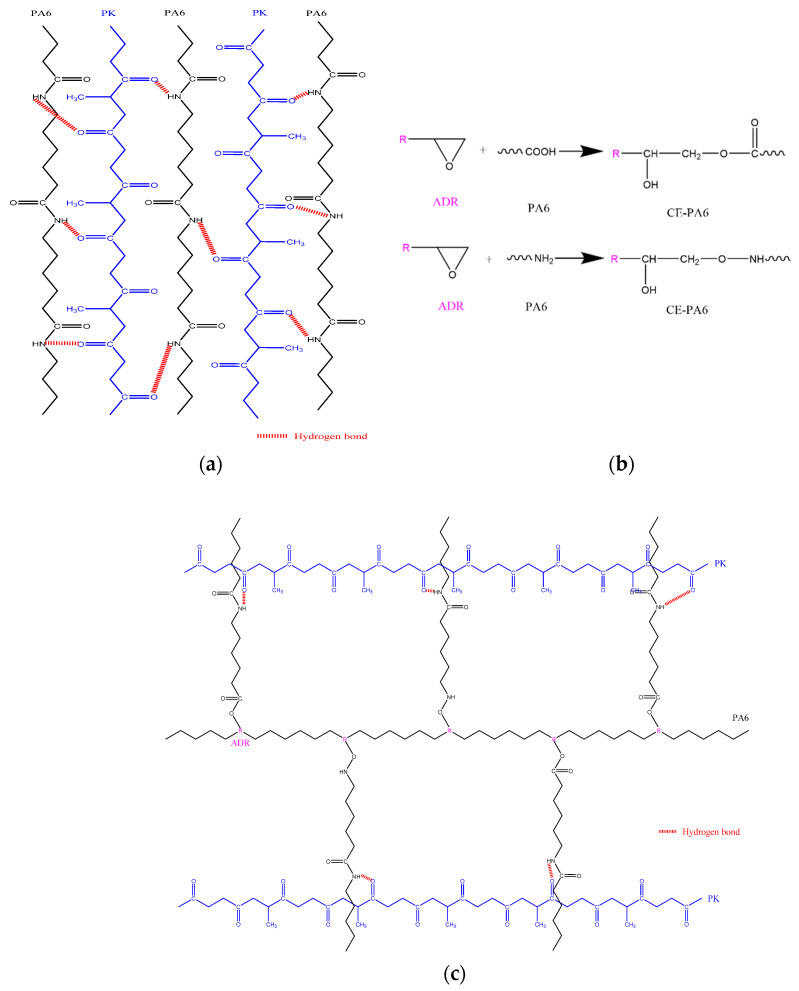
Schematics of chain extension: (**a**) PA6/PK blends; (**b**) PA6 with ADR; (**c**) PA6/PK blends with ADR.

The change in the torque of the mixer along with the mixing time of PA6 and PK at 220 °C is shown in Figure 1a. As can be seen in the figure, the torque increased rapidly initially when PA6 was fed into the mixer as it started to melt but stabilized within 2 min on melting. Once the torque stabilized after the addition of PK, the torque was higher compared with that of PA6 alone, which was due to the higher melt viscosity of PK. Another significant observation was that after 6 min, when PA6 and PK were substantially mixed, the torque again increased. The rapid increase in torque in the molten state suggests that reaction or mutual interaction was occurring. As PA6 and PK both have polar groups such as –NH and –C=O, we can expect hydrogen bonding between the polar groups in the PA6 domain and PK co-domain in the blend, once mixing occurs to a certain degree (Figure 1a). The schematic is shown in Figure 2a. Figure 1b shows the change in torque with time when 5 phr of the commonly used chain extender Joncryl ADR was added to PA6 prior to the addition of PK. Compared with the PA6/PK blend in Figure 1a, the torque initially did not show much difference when ADR was added prior to mixing PK; however, after a relatively short mixing time of 4 min, a drastic torque change occurred due to the interaction of the polar groups, with the increase being higher at higher PK contents. In addition, the time at which the torque started to increase decreased with increase in the PK content. This shows that the epoxy groups of the chain extender ADR and –COOH or –NH_2_ groups of PA6 reacted to form branches on PA6 (Figure 2b), and then physical crosslinks were formed between the branched PA6 and PK chains through hydrogen bonding interaction (Figure 2c).

**Figure 3:**
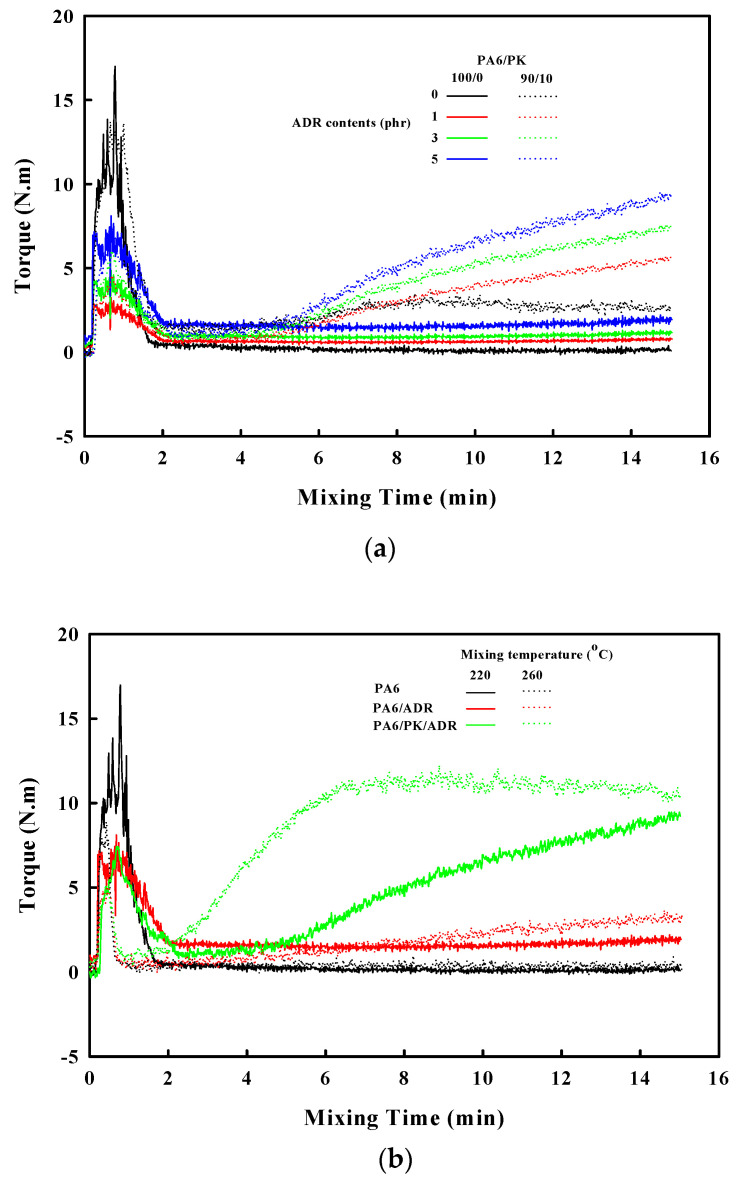
Effect of mixing time on the melt torque of PA an PA6/PK(90/10) blend (**a**) at different ADR contents and (**b**) processed at different mixing temperatures.

Figure 3a shows the effect of ADR content on the torque change in PA6 and PA6/PK (90/10) blend during the mixing process. In the case of PA6, a slight increase in the torque was observed with the increase in the ADR content due to chain extension, but a drastic increase in the torque was not observed with mixing time. However, in the case of the PA6/PK blend, a drastic increase in the torque was observed with increase in the ADR content. This suggests that with increase in the ADR content more branches occurred on PA6 due to chain extension, and the branched PA6 chains affected the hydrogen bonding with PK to change the resulting structure, that is, the structure was not the linear PA6 structure in Figure 2a, but rather the structure with physical crosslinks shown in Figure 2c. The effects of ADR content and mixing temperature on the change in torque in PA6 and PA6/PK blends are shown in Figure 3. Increase in the ADR content in PA6 increased the content of reactive ADR epoxy groups to increase the number of branches resulting in overall increase in the torque. However, in the case of PA6/PK blends, increase in the ADR content resulted in a drastic increase in the torque due to an increase in hydrogen bonding between the branches on PA6 and PK. The effect of mixing temperature on the change in torque was negligible in the case of PA6 due to its relatively low melt viscosity, but when ADR was added, the torque increased with increase in the mixing temperature, and when ADR and PK were added, the torque increase became more drastic and the time at which the torque increases became shorter. This suggests that the increase in the mixing temperature resulted in an increase in the formation of end groups with which the added ADR could react to form branches and in a drastic increase in the torque through hydrogen bonding between the branched PA6 chains and PK.

**Figure 4:**
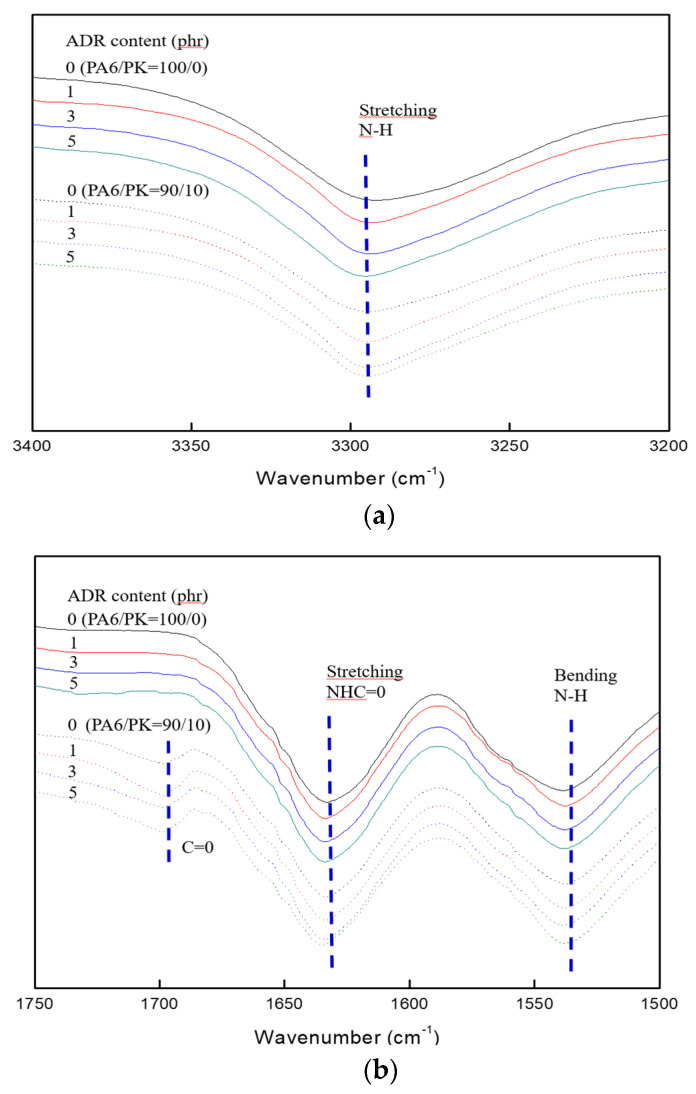
FTIR spectra of PA6 and PA6/PK blend (90/10) of different ADR contents: (**a**) N–H stretching (3294 cm^−1^); (**b**) C=O (1699 cm^−1^), NHC=O stretching (1634 cm^−1^), NHC=O stretching (1634 cm^−1^), N–H bending(1538 cm^−1^).

**Figure 5:**
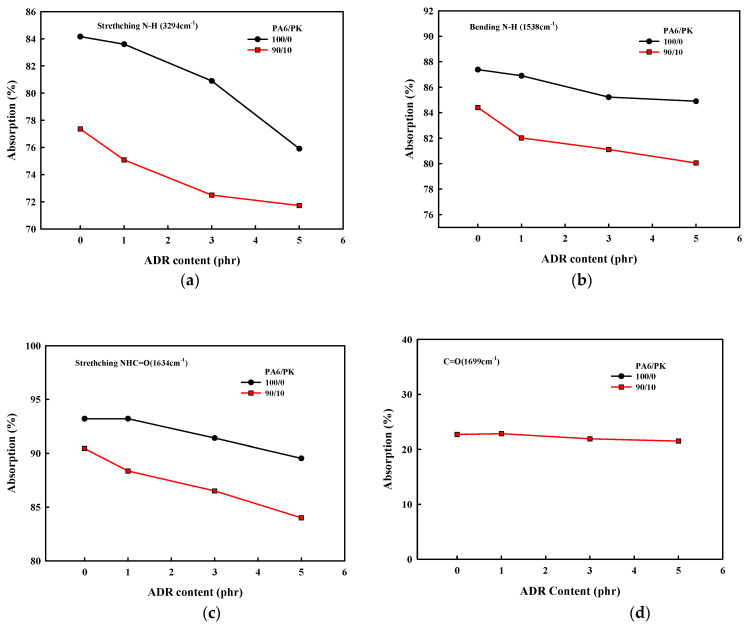
Effect of ADR content on the maximum absorption of the peaks due to amino and carbonyl groups in PA6 and PA6/PK blends:(**a**) N–H stretching (3294 cm^−1^); (**b**) N–H bending (1538 cm^−1^); (**c**) NHC=O stretching (1634 cm^−1^); (**d**) C=O (1699 cm^−1^).

The change in the FT-IR spectrum with addition of the chain extender ADR in PA6 and PA6/PK 90/10 blend is shown in Figure 4, and the change in the maximum absorption of the respective peaks with the amount of ADR added is shown in Figure 5. The change in the maximum absorption of the characteristic FT-IR peaks of the amine N-H stretching peak at 3294 cm^−1^and the bending peak at 1538 cm^−1^ of PA6 with ADR content are shown in Figure 5a,b, and the change in the maximum absorption of the FT-IR peak of the NHC=O bond at 1634 cm^−1^ and C=O bond at 1699 cm^−1^ in PK and PA6/PK blends are shown Figure 5c,d, respectively. There was no change in the maximum absorption of the C=O bond in PK with ADR content, while the maximum absorption of the –NH bond and NHC=O bond of PA6 decrease with the addition of PK and ADR. This shows again that the addition of ADR results in a change in the chain structure from the formation of branches and that a change in the chain structure due to hydrogen bonding between the polar C=O of PK and the NH– and –NHC=O of PA6 is occurring simultaneously.

**Figure 6:**
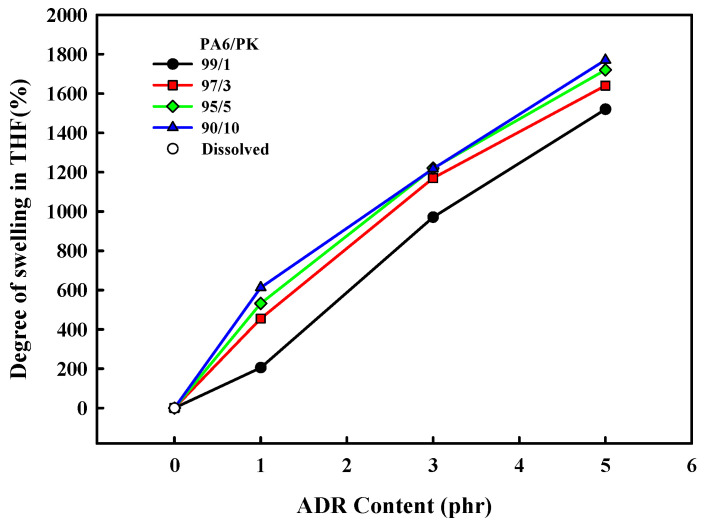
Effect of chain extender, ADR content on the degree of swelling of PA6/PK blends swollen in THF at 50 °C for 100 min.

**Figure 7:**
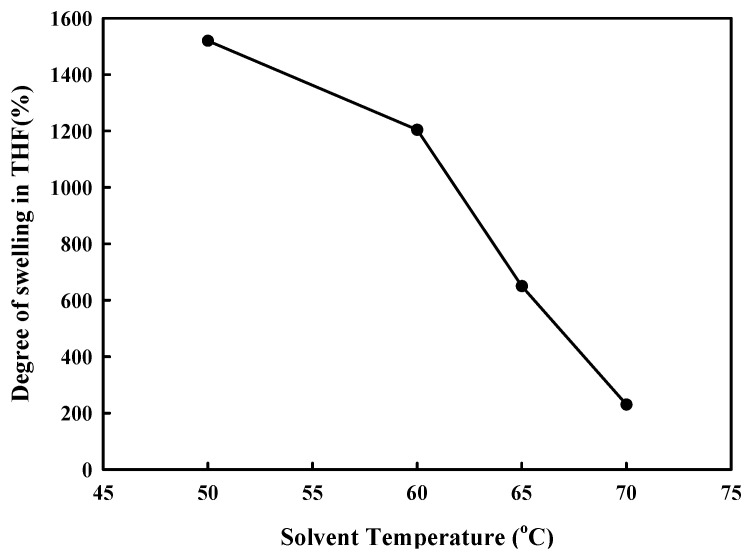
Effect of solvent temperature on the degree of swelling of PA6/PK(90/10) blend with ADR in THF for 100 min.

To confirm the change in the chain structure due to hydrogen bonding between PA6 and PK, the change in the degree of swelling of the PA6/PK blend THF at 50 °C with ADR content is shown in Figure 6. The PA6 and PA6/PK blend dissolved completely in THF in the absence of ADR, and PA6 dissolved in THF even when ADR was present, but when ADR was added to the PA6/PK blends, they did not dissolve in THF but just swelled. The degree of swelling of the PA6/PK blend increasedwith the ADR content and amount of PK in the blend. This result suggests that the change in the chain structure on addition of ADR to PA6 and to PA6/PK blend is different. That is, chain extension of PA6 with ADR resulted in branched PA6 (Figure 2b), which is soluble in THF, while the addition of ADR to PA6/PK blends resulted in physical crosslinking between the branched PA6 chains and PK through hydrogen bonding (Figure 2c), and thus they did not dissolve in THF but swelled. The increase in ADR content increased the degree of branching of PA6, and thus, the amount of hydrogen bonding with PK to increase the degree of swelling. To observe the effect of dissolution temperature on the solubility of PA6 in THF, the change in the degree of swelling with solvent temperature is shown in Figure 7, where the sample was the PA6/PK 90/10 blend in which physical crosslinking through hydrogen bonding was expected. If the crosslinking was chemical, the degree of swelling should increase with solvent temperature; however, the degree of swelling decreased and there was also a change in the shape of the sample suggesting an increase in the solubility results in partial dissolution of the ADR containing PA6/PK blend. This substantiates the observation that the crosslinking between branched PA6 and PK in the presence of ADR was not chemical but physical crosslinking.

**Figure 8:**
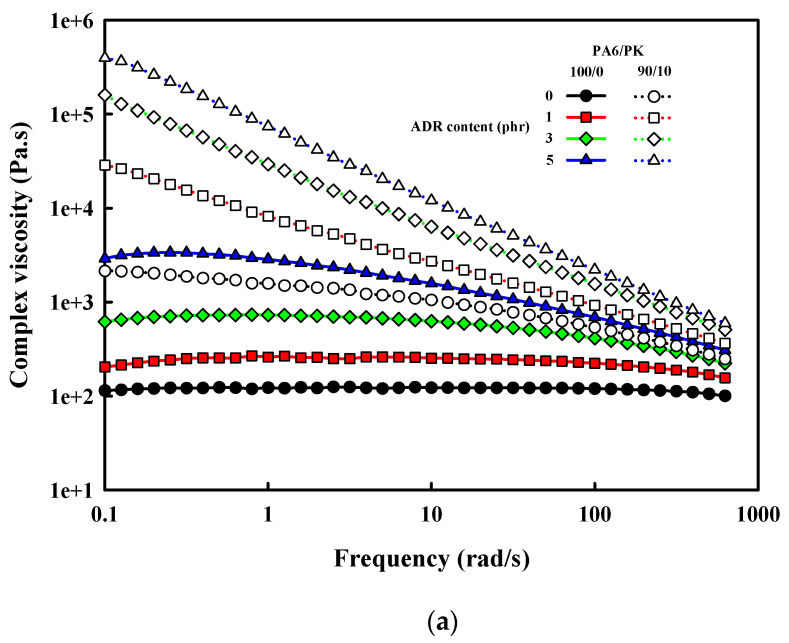

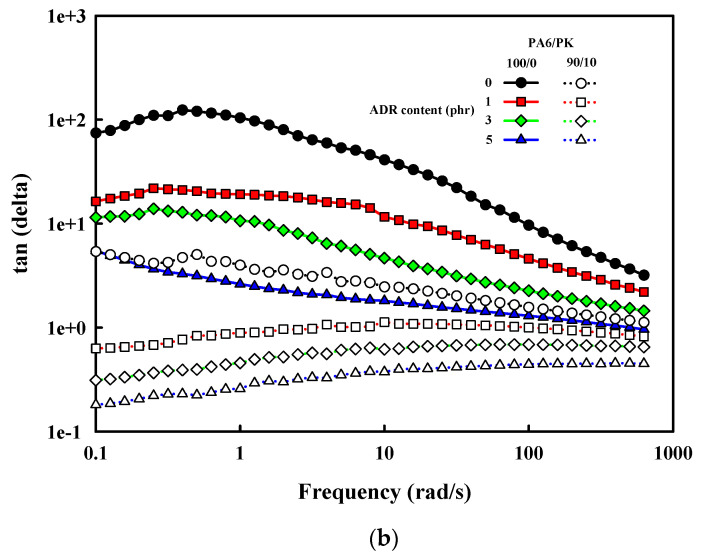
Rheological properties of PA6 and PA6/PK blend with chain extender, ADR: (**a**) complex viscosity; (**b**) loss tangent.

**Figure 9:**
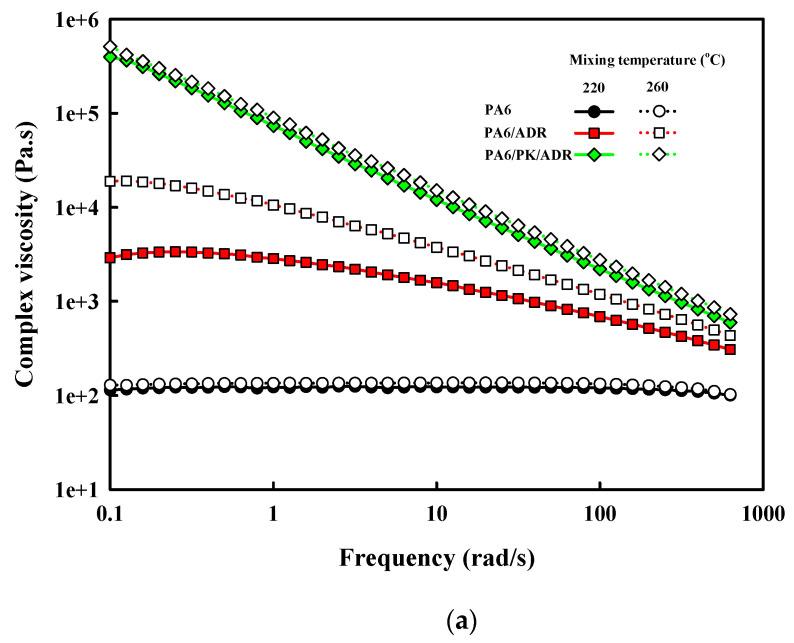

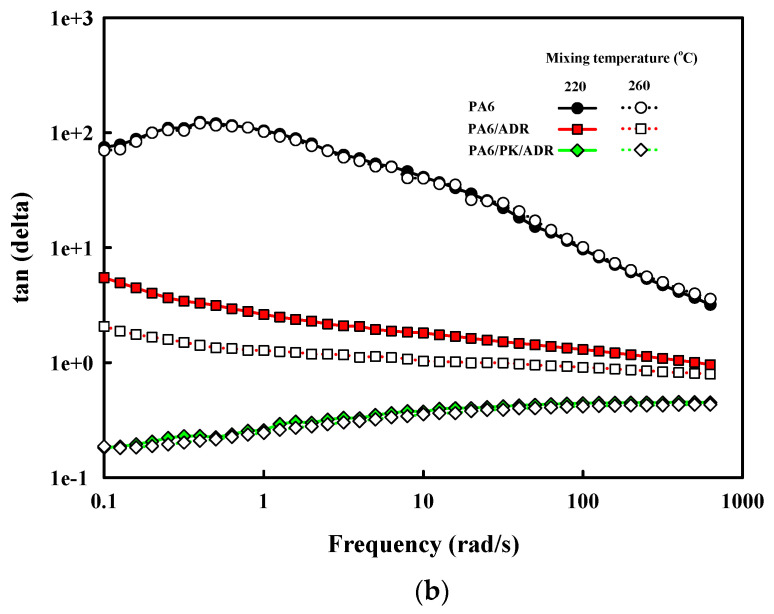
Effect of mixing temperature on the rheological properties of PA6 and PA6/PK blend with chain extender, ADR: (**a**) complex viscosity; (**b**) loss tangent.

The dynamic rheological properties are known to be greatly affected by branching and crosslinking [30]. The complex viscosity and the loss tangent of PA6 and PA6/PK blend shown in Figure 8 show that both exhibited a typical non-Newtonian shear thinning behavior and that the addition of ADR resulted in an increase in the viscosity with the shear thinning behavior becoming more pronounced with increase in the ADR content. The effect of chain branching on the shear viscoelasticity is generally known to be dependent on the length of the branch [31]. Short branches cause a small change in the viscosity and a small change in viscosity with shear rate as they are within the entanglement radius of the melt, while long branches protrude out of the entanglement radius to increase the viscosity and accentuate the decrease in the viscosity with increase in shear rate. The loss tangent shown in Figure 8b also decreased with the formation of long chain branches in the presence of chain extender. The loss tangent has an inverse relationship with the melt strength, which affects the melt processing properties [32]. That is, the melt processability of PA6 was enhanced by the addition of ADR due to the formation of long chain branches. In the case of PA6/PK blends, the complex viscosity increased due to the relatively high viscosity of PK and the suggested hydrogen bonding between PA6 and PK, exhibiting a drastic increase with addition of ADR, and the shear thinning behavior characteristic of non-Newtonian fluids also showed a more drastic change compared with when only ADR was added to PA6. This shows that the change in chain structure due to ADR was completely different in the case of PA6 and PA6/PK blends as shown in Figure 2. As seen in Figure 6 and Figure 7, contrary to the formation of long chain branches with the addition of ADR to PA6, the hydrogen bonding between branched PA6 and PK formed physical crosslinks, which caused a drastic increase in the viscosity and shear thinning behavior in the case of PA/PK blends. The change in the viscoelastic properties with these changes in the chain structure resulted in a significant decrease in the loss tangent at low shear rates, as can be seen in Figure 8b. Thus, minimization of the decrease in the molecular weight through chain extension and the formation of physical crosslinks between branched PA6 and PK may also solve the low melt viscosity drawback in PA6 melt processing. The effect of mixing temperature on the rheological properties of PA6/PK blends shown in Figure 9. PA6 did not exhibit a significant change in the viscosity with the change in mixing temperature from 220 to 260 °C, suggesting that there was not a significant change in the extent of thermal degradation at these temperatures. When ADR was added to PA6, an increase in viscosity occurred. This suggests that although there was not a significant decrease in the molecular weight, more end groups were formed that reacted with ADR to form branches increasing the viscosity. However, the loss tangent increased, suggesting there was a change in the length of the branches with change in mixing temperature. In the case of PA6/PK blend with added ADR, the mixing temperature did not have an effect on the melt viscosity or the loss tangent, suggesting that the change in the chain structure with change in mixing temperature did not have a significant effect on their rheological behavior. This suggests that the physical crosslinking through hydrogen bonding had a greater effect on the rheological properties compared with that of the branching of PA6.

**Figure 10:**
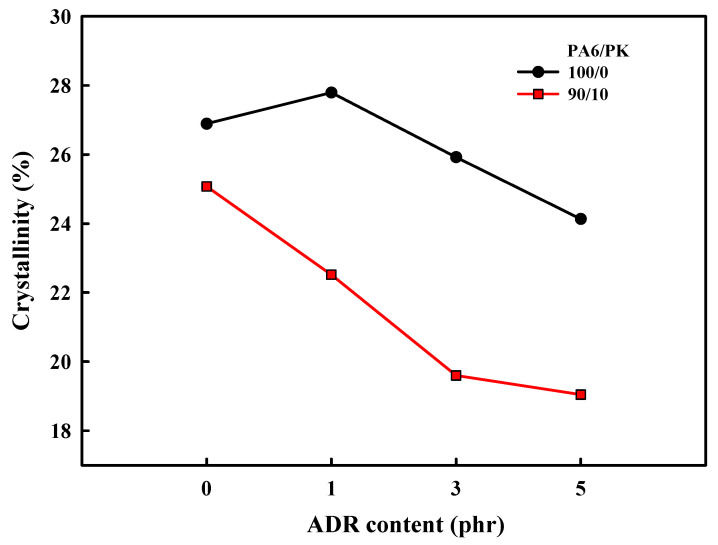
Effect of chain extender, ADR, content on the crystallinity of PA6 and PA6/PK blends with chain extender, reflecting the influence of chain branching.

**Figure 11:**
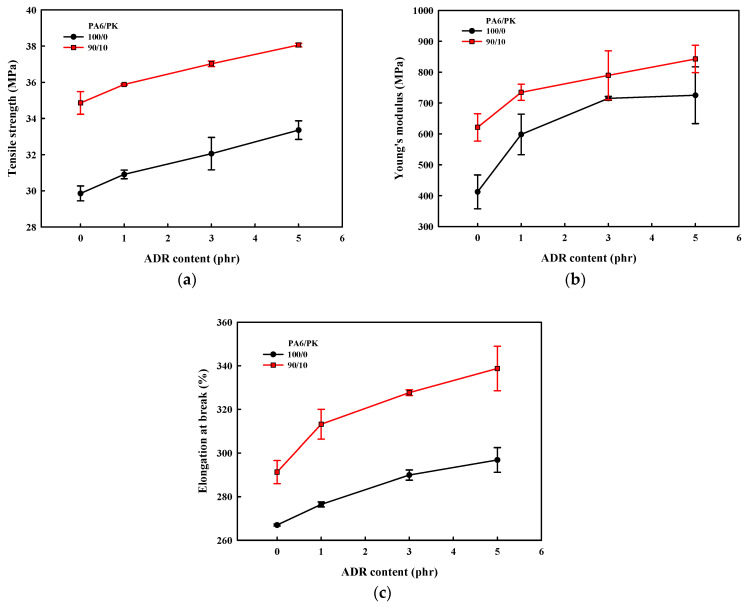
Effect of chain extender, ADR, content on the mechanical properties of PA6 and PA6/PK blends with chain extender, reflecting the influence of chain branching: (**a**) tensile strength; (**b**) Young’s modulus; (**c**) elongation at break.

The change in the crystallinity with the addition of ADR to PA6 and PA6/PK blends is shown in Figure 10. The crystallinity of PA6 decreased on blending with PK and that of PA6 and PA6/PK blends all decreased with the addition of ADR. The decrease in crystallinity with addition of ADR was due to the change in the chain structure caused by the chain extension with ADR. As confirmed, the long chain branching and the physical crosslinking effected by addition of ADR and PK decreased the regularity of the chain and hindered the crystallization of nylon 6 to decrease its crystallinity. The greater decrease in the crystallinity on addition of ADR to PA6/PK blends compared with PA6 suggests that physical crosslinking with addition of PK decreased the crystallinity to a greater extent compared with long branching formed by chain extension with ADR. The crystallinity also decreased when mixed at higher temperature and with an increase in the ADR content, suggesting that the degree of branching of PA6 with ADR also influenced the crystallization of PA6. The changes in the mechanical properties of PA6 and PA6/PK blends with ADR content in Figure 11 show that the mechanical properties were enhanced by the addition of ADR and PK. The change in chain structure by the introduction of long branches and physical crosslinks through the addition of ADR increased the melt viscosity to enhance the melt processability, which also increased the chain orientation. Increase in chain orientation is known to increase the physical properties of polymers such as tensile strength and elastic modulus. Especially, physical crosslinking exhibits a greater increase in the elongation at break compared with long chain branching, as observed in previous figures.

## 4. Conclusions

Chain extenders used widely in the melt processing of polymers and PK capable of hydrogen bonding were added in the melt processing of PA6, and their effects on the chain structure and properties of PA6 were studied. Long chain branching was formed on PA6 through the addition of the chain extender ADR, and physical crosslinking was introduced by further addition of PK, which formed hydrogen bonds with long chain branched PA6. These changes in the chain structure increased the melt viscosity of PA6 and accentuated the shear thinning behavior to enhance the melt processability. Especially, the physical crosslinking introduced by the addition of PK was effective in the enhancement of the melt processability of PA6 and also resulted in a decrease in the crystallinity and an increase in the physical properties.

## Data Availability

Data are in the authors’ possession.
